# AZALEP a randomized controlled trial of azathioprine to treat leprosy nerve damage and Type 1 reactions in India: Main findings

**DOI:** 10.1371/journal.pntd.0005348

**Published:** 2017-03-30

**Authors:** Diana N. J. Lockwood, Joydeepa Darlong, Pitchaimani Govindharaj, Royce Kurian, Pamidipani Sundarrao, Annamma S. John

**Affiliations:** 1 London School of Hygiene & Tropical Medicine, London, United Kingdom; 2 Purulia Leprosy Mission Hospital, Purulia, India; 3 Leprosy Mission Trust India, Noida, India; University of California San Diego School of Medicine, UNITED STATES

## Abstract

**Background:**

Leprosy Type 1 reactions are difficult to treat and only 70% of patients respond to steroid treatment. Azathioprine has been used as an immune-suppressant and we tested its efficacy in treating leprosy T1R.

**Methodology:**

Randomised controlled trial adding azathioprine to steroid treatment for leprosy reactions. This trial was conducted in four leprosy hospitals in India. Patients with a new leprosy Type 1 reaction affecting either skin or nerve were recruited. They were given a 20 week course of oral prednisolone either with placebo or azathioprine 50mg for 24, 36 or 48 weeks. Outcomes were measured using a verified combined clinical reaction severity score (CCS) and the score difference between baseline and end of study calculated. An intention to treat analysis was done on the 279 patients who had an outcome.

**Principal findings:**

345 patients were recruited, 145 were lost due to adverse events, loss to follow up or death. 36% needed extra steroids due to a recurrence of their skin and/or nerve reaction. 76% of patients had improvements in their CCS the end of the study, 22% had no change and 1.1% deteriorated. Adding azathioprine to steroid treatment did not improve CCS. So the improvements were attributable to treatment with steroids. We analysed the skin, sensory and motor scores separately and found that skin improvement contributed most with 78.9% of patients having skin improvement, azathioprine treatment for 48 weeks improved sensory scores it also improved motor scores but so did treatment with prednisolone alone. We identified significant adverse effects attributable to steroid treatment. When azathioprine and Dapsone were given together significant numbers of patients developed significant anaemia.

**Conclusions:**

Azathioprine is not recommended for the treatment of leprosy reactions and does not improve steroid treatment. Recurrent reactions are a major challenge. We have also identified that 65% of patients with sensory and 50% with motor nerve damage do not improve. Future studies should test giving azathioprine in the treatment of nerve damage and giving a higher dose for 48 weeks to patients. These findings highlight the difficulty in switching off leprosy inflammation and the need for better treatments for reactions and nerve damage. There is also a research need to identify patients who have recurrences and optimize treatments for them. Patients with recurrences may benefit from combined treatment with steroids and azathioprine. We have also shown that significant numbers of patients treated with steroids develop adverse effects and this needs to be highlighted in leprosy programmes. Research is needed to identify patients who do not respond to steroid treatment and develop alternative treatments for them.

**Trial Registration:**

ClinicalTrials.gov This trial was registered with the Indian Council of Medical research clinical Trial register as a clinical trial Number—REFCTRI/2016/12/007558

## Author summary

Type 1 reactions affect leprosy patients and are due to increased inflammation in skin and nerves that can cause disfiguring skin lesions and loss of sensation and loss of power in the hands and the feet. These disabilities can lead to deformity and severe disability. It is important to improve the treatment for T1 reactions. T1R are currently treated with steroid tablets and about 50% patients will have improvement in their nerve function after treatment. Azathioprine is a cheap widely available immune-suppressant and we tested whether it could improve skin and nerve function in leprosy patients. We did a randomised double blind study in four leprosy hospitals in India giving 345 patients treatment with steroids plus either azathioprine or placebo. 78% of patients had improved skin, 35% had improved sensory and 50% had improved motor nerve function at the end of treatment. Treatment with azathioprine did not increase patient improvement and the improvements we found were associated with steroid treatment. There was a high rate of adverse effects from both steroids and azathioprine. These findings highlight the difficulty in switching off leprosy inflammation and the need for better treatments for reactions and nerve damage. The problems of steroids causing adverse effects in patients needs to be highlighted in leprosy programmes. Research is needed to identify patients who do not respond to steroid treatment and develop alternative treatments for them.

## Introduction

Leprosy is complicated by immune–mediated reactions which affect about 30% of patients with Borderline leprosy.[1] These manifest clinically with inflammation of skin lesions and neuritis (nerve inflammation) which produces loss of sensory and motor nerve function. They are important because the loss of nerve function can lead to long term disability and the inflamed skin lesions are disfiguring. The underlying pathology of these inflammatory episodes is increased delayed type hypersensitivity to *M leprae* antigens with CD4 cells in lesions and activated macrophages and oedema. T1Rs are mediated via Th1 type cells and the pro-inflammatory cytokines, IL12 TGF b and the oxygen-free radical producer iNOS are all found in lesions.[2, 3] Reactions may occur before, during or after anti-bacterial Multi Drug Therapy.

There are few good randomised controlled trials on the effectiveness of steroids in the treatment of T1R, and a Cochrane Review only included three trials.[4] Steroids are widely used in leprosy reactions and most skin lesions improve with treatment but nerve function improvement is less satisfactory, with a median improvement rate of 50% (Range 30–86%). An RCT done in India comparing three different regimens found better outcomes with 20 versus 12 weeks of treatment, whereas the outcomes with different starting doses 60 mg or 30 mg were similar. [5]

Alternatives to steroid treatment are needed because of the high relapse rate for T1R [6] and patients having contraindications to steroid treatment such as diabetes, an increasing problem in India. Alternative treatments are also needed for patients with reactions who do not improve on steroid treatment and those who become steroid dependent.

Since the underlying pathology is one of immune mediated inflammation we hypothesised that adding in additional immuno-suppression would improve outcomes. Azathioprine is an immuno suppressant used in immune mediated diseases.[7] It is metabolized to mercaptopurine by the enzyme thiopurine methyltransferase which inhibits T cell development.

A trial in Nepalese leprosy patients with T1R compared a 12 week Azathioprine Prednisolone combination versus prednisolone alone and found the dose of prednisolone needed was less in those on the combined regimen. [8] One of the shortcomings of the Nepal study was that the azathioprine was only used for 12 weeks which is insufficient time to assess clinical efficacy. We therefore wanted to test the efficacy of azathioprine over a longer period.

Adverse effects due to azathioprine may occur in up to 15% of patients and include nausea, vomiting and mucosal ulcers and bone marrow suppression.[9] Careful clinical monitoring of adverse effects of azathioprine is needed, especially in resource poor settings.

We tested our hypothesis that azathioprine would be more efficacious than prednisolone alone by conducting a four arm randomised controlled trial. This study was done in four leprosy hospitals in north India; patients with T1R and/or neuritis were recruited and given a 48 week course of treatment. This comprised either a 20 week course of prednisolone alone, or prednisolone plus azathioprine for either 24, 36 or 48 weeks in a double blind design.

The objectives of the trial were therefore to determine whether the addition of Azathioprine to Prednisolone

1. Improves dermatological outcomes in Type 1 reactions.
2. Improves neurological outcomes in Type 1 reactions and neuritis
3. Reduces the recurrence rate (i.e. requirement for further treatment with steroids) in patients with T1R and neuritis.
4. To compare the effectiveness of different duration of treatment with Azathioprine in T1R and neuritis.

The outcome measures were skin inflammation measures and neurological measures which assess sensory and motor function in peripheral nerves. We used scales for assessing skin lesions developed for a previous trial on T1R.[10]

## Materials and methods

### Study centres

Four TLM (The Leprosy Mission) centres participated in this study: The Leprosy Mission Community Hospital, Shahdara, New Delhi; The Leprosy Mission Hospital, Faizabad, Uttar Pradesh; The Leprosy Mission Hospital Champa, Chattisgarh; and Purulia Leprosy Home and Hospital, West Bengal. The trial started in Aug 2007 and finished in 2012.

### Intervention

All patients received a 20 week course of prednisolone (P) as follows: P was started at a dose of 40 mg per day and reduced by 5 mg/day every two weeks till a dose of 20 mg/day was reached; 20 mg/day was given for four weeks, then 15 mg/day for four weeks. After this 10 mg/day P was given for two weeks and 5 mg/day for two weeks, (40x2, 35x2, 30x2, 25x2, 20x4, 15x4, 10x2 and 5x2 = 20 weeks). Patients were randomised to receive concomitant medication for 48 weeks with azathioprine (A) 50 mg fixed dose for 24, 36 or 48 weeks (APC) or a placebo. Each active treatment arm was made up to 48 weeks with placebo for 28, 24, or 12 weeks, so all patients took 48 weeks of medication.

The medication was prepared by and stored at The Leprosy Mission Research Centre, Delhi. Numbered treatment packs were made up and delivered to the centres. Specially designed forms were used for each stage of the recruitment, consent, assessment, laboratory investigations and follow-up process.

### Case definitions, inclusion and exclusion criteria: [6]

The following patient based definitions were used

**Paucibacillary (PB) leprosy—**when a patient had five or fewer well-demarcated, hypo-pigmented and anaesthetic skin lesions, and/or one peripheral nerve thickened.

**Multi bacillary (MB) leprosy—**when a patient had more than five skin lesions and/or more than one nerve involved.

**Skin Type 1 Reaction—**when patient had erythema and oedema of skin lesions.

**Nerve Function Impairment (NFI)—**present when a patient had new sensory impairment, motor impairment or both. Nerve pain alone was not included in the definition of neuritis.

**New nerve function loss–**is ‘New’ when it occurred within the previous six months. This was established by asking the patient about symptoms and duration of nerve function loss.

**Old nerve function loss—**is ‘Old’ when it started six months or more before the date of presentation. This was established by asking the patient about symptoms and duration of nerve function loss.

**Motor function loss**—is present when there was a muscle score below five in any of the muscles on voluntary muscle testing. The nerves tested were facial, ulnar, median, radial and lateral popliteal with the following muscles/movement strengths: orbicularis oculi, abductor digitii minimi, abductor pollicis brevis, wrist extension, tibialis anterior.

**Sensory function loss**—is defined as a loss of two or more sensory points on Semmes Weinstein testing in the area supplied by a sensory nerve. Sensory testing was done for ulnar, median and posterior tibial nerves.

**Worsening of motor function–**is defined as a change in one or more than one grade in the voluntary muscle testing.

**Worsening of sensory function—**is defined as a change in two or more than two points in the sensory testing.

**Nerve recovery—**is when the NFI scores regain normal values for either sensory or motor functions.

**Nerve improvement—**is when the NFI scores improve from the baseline value for either sensory or motor functions but does not regain normal values.

**Most severely affected nerve—**is a nerve with a score of 3 on motor testing and /or 1.5 on sensory testing.

**Least affected nerve**—is a nerve with a score of 1 on motor testing and/or 0.5 on sensory testing.

**Recurrent reactional skin lesions—**are defined as newly erythematous lesions that have either developed whilst on steroids or when patient has stopped steroids.

**Restarting steroids–**is restarting or increasing steroid therapy during the study in the presence of worsening nerve function or recurrent skin reactions as defined above.

**Defaulter–**is a patient who did not return to the hospital within four weeks of the last visit and so is removed from the study.

**Withdrawn–**is when a patient is removed from the study due to adverse effects.

### Primary outcome

Improved combined score- measured by skin, motor and sensory scores.

### Secondary outcomes

1. Prevention of recurrent skin reactions.
2. Prevention of recurrence of neuritis during or after treatment

### Measurement of sensory function

Sensory testing (ST) was performed using Semmes-Weinstein monofilaments (SWM) (Sorri-Bauru, Bauru, São Paulo, Brazil) at designated test sites on the hands and feet.[11] The sensation in the areas of skin supplied by the ulnar and median nerves was tested with 2g SWM at six sites. Sensation on the feet was tested using 10g SWM at four sites. For both sets of sensory testing a score was developed. For each nerve the score ranged from 0–1.5 depending on the number of sites at which the monofilament was perceived. The range of scores for each foot was (0–1.5).The sensory scores were summated on every visit. *(See Appendix 1)*.

A similar method of assessment was used in a published study. [6]A score of 0 for an individual nerve indicated that complete sensation was present and a score of 1.5 that there was no sensation in the area supplied by that nerve. A patient with a sensory score of 3 either had slight impairment at several points in different nerves or two nerves with complete loss of sensation. A modification of the Walker scale was needed because the two studies used different sets of monofilament for sensory testing. In the AZALEP study 2 and 10 gram monofilaments were used on the hands and feet respectively whereas in the Walker study 2 and 10 gram monofilaments were used on the hand, 10 and 300 gram monofilaments were used on the feet.

### Measurement of motor function

Voluntary muscle testing (VMT) was assessed using the modified Medical Research Council (MRC) grading of power. The grading was modified in our scores: a MRC grade 5 = 0, 4 = 1, 3 = 2 and 2, 1 and 0 were grouped together as 3. The scores range from 0–3 for each muscle with a total range of 0–30. The scores for each muscle were summated on each visit. A score of 0 indicated completely intact muscle power and 30 would indicate severe weakness in all the muscles tested. A score of 9 could indicate loss of function in three muscles or slight weakness in all muscles **(See supporting Information).**

#### Nerve Score

The sensory and motor scores were totaled.

#### Skin Score

The severity of the reactional skin lesions was assessed using a previously validated score (Walker 2009) which assessed the skin associated domains: degree of inflammation, percentage of lesions inflamed, and peripheral oedema on a 0–3 point scale. The score range was 0–9. A score of zero indicated no skin lesions and a score of 9 occurred with severe skin lesions **(See supporting Information).**

### Combined score

The skin, sensory and motor scores were summated for each patient on each visit. Trial outcomes were assessed by looking at score differences.

For each individual nerve the presence of new nerve damage was determined from the patient’s history and VMT/ST. New nerve damage had to be present for the patient to be entered into the study. Old nerve damage was separately recorded because it was not expected that these nerves would improve.

### Inclusion criteria

Patients with PB and MB leprosy were included if they had

- acute neuritis,
- reactional skin lesions,
- or both the above

Patients already on steroid therapy (started within the past four weeks) were included if they presented with new neuritis. Patients taking or having completed MDT were eligible.

### Exclusion criteria

The following exclusion criteria (all defined in the protocol) were applied: age less than 15 years or more than 60 years, weight less than 30 kg, confirmed pregnancy, on treatment for tuberculosis, known HIV sero-positivity, hepatic dysfunction, bone marrow dysfunction, splenomegaly, hypertension and diabetes, Erythema Nodosum Leprosum (ENL) reactions. Patients unable to comply with monitoring and follow up requirements were also excluded.

All patients had a full blood count (haemoglobin, white cell, differential and platelet counts), renal function test (serum creatinine), liver function tests (SGOT & SGPT) and a random blood sugar done to detect underlying conditions for exclusion.

### Ethical approval

Ethical approval for this study was obtained from both the London School of Hygiene & Tropical Medicine, London UK and the Ethics Committee of The Leprosy Mission Trust India on 8th February 2008.

This trial was registered with the Indian Council of Medical research clinical Trial register as a clinical trial Number—REFCTRI/2016/12/007558

Informed Consent was taken from patients who were eligible to be included in the study.

All eligible patients were invited to participate in the study by the study Medical Officer (MO). They received a detailed information sheet (Form 1.0) about the Azathioprine study (in the local language); a patient counsellor helped them understand the study details. The patients then had the above investigations. Those found eligible returned to the study MO, where they gave their signed informed consent (Form 2.0) to participate in the study, and were included.

Patients who were either ineligible for trial entry or who did not wish to participate were treated as per the routine practice in that hospital. Patients were also informed that if they did not consent, this would not affect their usual treatment.

### Sample size calculation

The sample size calculation was based on the expected improvements in the combined scores (skin, sensory & motor) that the addition of azathioprine to prednisolone would produce. It was calculated that the addition of azathioprine would produce a 25% improvement in the combined scores. This would be a score of 4 allowing for an alpha of 0.05 and beta of 0.80, the minimum sample size is calculated as follows:

n=2x[(Zα+Z1-β)xSD)/d]2=2x[(1.96+0.842))x2)/1]2=63pergroup.

We assumed a ‘lost to follow up’ rate of 15%, and dropout rate due to adverse reactions of 10%, so minimum of 78 patients would be required per arm, and a total of 312.

### Randomisation & allocation to treatment arms

The staff at the centres were blinded to the randomisation. The Head of the Research Resource Centre (RRC), using Random Sampling Numbers prepared the confidential allocation into four arms, which was balanced for each centre, and created lists with an unique serial number. The allocation for each patient was kept confidential at the RRC.

The packing was done at RRC in small daily pill boxes with the appropriate amounts of each drug for each arm for four weeks. The boxes were prepared for 48 weeks for each patient. Thus there were 12 boxes for each patient, distributed to the respective centres by courier.

### Drug preparation

The drugs were stored in cool dry rooms both at RRC and at the centres. Drugs were ordered in batches to ensure that they were within the active period after manufacture.

The placebo tablet was identical to the Azathioprine tablet.

Azathioprine Tablets BP 50 Mg (TRANSIMUNE*) and the placebo tablets were manufactured by Troikaa Pharmaceuticals Ltd. Thol-382 728, Gujarat, India.

**Prednisolone Tablets I.P** were manufactured by Comprehensive Medical Services India, (Essential Drugs Project) Riverside, P.B.No.988, Manapakkam, Chennai -600 089.

### Recruitment

Physiotherapists in each hospital identified new eligible patients. The patient had clinical and laboratory investigations according to the flow chart (Appendix 2) and the clinical history with details of skin type 1 reaction, nerve function impairments and other clinical details was recorded. The pre-treatment assessment form was filled in; they were given a box with four weeks treatment and a date for the follow up visit.

### Follow up and monitoring

Patients were followed up fortnightly for the first eight weeks and then at four week intervals, with full clinical and neurological assessments and laboratory investigations done at each visit.

If the scores had worsened, the patients were observed for seven days, with repeat testing and the protocol for increasing prednisolone was followed. Compliance with the medication was recorded. Possible adverse effects were detected by relevant questioning and investigations.

Any participant who did not return for a follow up for more than two weeks was contacted at his / her home by phone or personal visit and invited to return. Any patient who returned to the hospital within four weeks of the last visit continued in the study and those who did not return were classed as defaulters.

In patients who developed infections during the first 20 weeks, their dose of prednisolone was tapered more quickly (decreasing by 5mg every three days) and stopped, and antibiotic therapy relevant to the presumed infection given. In patients who were on MDT and developed anemia, dapsone was stopped. If the adverse events resolved within four weeks, the patient was restarted on the drugs and continued in the study. If this period exceeded four weeks or the adverse event was severe the patient was withdrawn from the study.

### Criteria for restarting or increasing prednisolone

If the neuritis worsened or there was a recurrence of type 1 skin reaction during the first 20 weeks, the dose of prednisolone was increased to the preceding dose resulting in a longer duration of steroid therapy. If worsening occurred after 20 weeks, when prednisolone treatment had finished, the patient was given a 12 week course of prednisolone, starting with 40mg as in the protocol (two weeks each of 40, 30,20,15,10 & 5 mg of prednisolone).

### Monitoring and management of adverse effects

Potential adverse effects of steroids, azathioprine and MDT were closely monitored. Protocols were developed for the investigation and management of all complications.

Adverse effects and deaths were discussed by the clinical team and ascribed when possible to one of the drugs. Deaths were investigated and the cause of death established by the MO. The contribution of Azathioprine and prednisolone to the cause of death were reviewed.

### Documentation

All the patients’ details were recorded in predesigned forms designed for the study, at baseline and at each subsequent visit. These covered *Screening for Eligibility*, *Registration for Trial*, *Informed Consent*, *Baseline History & Examination*, *Baseline Nerve Function Measurement*, *Baseline Lab Investigations*.

### Data management

Data from the clinical trial including the baseline data were computerised using dedicated software as well as on MS Excel sheets. Data was transferred into Statistical Package for Social Sciences and analysed.

The trial data is stored at the Leprosy Research Centre, Delhi. It can be obtained from the Head of the Leprosy Mission Research Centre, India, currently Dr annamma John.

### Statistical analysis

Chi-square testing was used to compare the variables such as age, sex, type of leprosy.

For each of the primary and secondary outcomes, the differences between the baseline and end of study were computed, for each arm and the paired t-test was used to determine the statistical significance. The actual p-value was noted and inferences made accordingly using the conventional 5% and 1% levels.

The differences between the patients receiving placebo and each of the three Azathioprine treatment arms were also tested for statistical significance using the independent t-test for the various outcomes.

A modified intention to treat analysis was done on the outcomes for all the patients entered in the study (345) excluding those who had an extra course of prednisolone. A per protocol analysis was done on the patients who took the complete course of treatment and did not receive additional prednisolone (134). Outcomes for skin, motor and sensory testing were compared for patients in the four treatment arms. Median improvement scores were calculated and the differences tested for statistical significance using the Kruskal-Wallis test. Survival curves using Kaplan-Meier method for recurrence events were calculated for each arm.

## Results

### Baseline analysis

Three hundred and forty-five patients (86.1% males and 13.9% female), age range 15 to 60 years, were recruited from the four centres.

Three hundred and thirty-six (97.4%) of the patients had Multi Bacillary leprosy and Ridley-Jopling types Borderline Tuberculoid (BT)62.3%, Borderline Borderline (BB) 5.5%, Borderline Lepromatous (BL) 21.7%, and polar Lepromatous (LL) 5.8%. 80% of the patients were starting MDT (40%) or on MDT (40%) and 20% had completed MDT.

Table 1 shows that 345 patients recruited had similar distributions of skin reaction and/or the presence of sensory and motor impairments and skin reaction in each centre and for each trial arm. Skin reaction alone (36.8%) was the commonest reason for recruitment, followed by new sensory and/or motor damage alone. Thirty-six(4%) had new nerve function impairment.

**Table 1:** Clinical Features, Sensory, Motor impairment and Skin Reaction by arm for 345 patients.

Profile At Enrollment	Arm (%)				Total (n = 345)
	1 (n = 87)	2 (n = 86)	3 (n = 88)	4 (n = 84)	
Reaction(R))	37.93	32.56	42.05	34.52	36.81
SM	17.24	17.44	14.77	23.81	18.26
Motor (M)	22.99	11.63	17.05	10.71	15.65
SMR	8.05	12.79	11.36	13.1	11.3
MR	6.9	11.63	4.55	10.71	8.41
SR	6.9	10.47	4.55	5.95	6.96
Sensory (S)	0	3.49	5.68	1.19	2.61
Total	100	100	100	100	100

Key: MR = Motor and reaction, SR = Sensory and Reaction, SM, Sensory and Motor, SMR = Sensory, Motor and reaction.

Bacterial Index: Smear negative -210 (62.5%), BI 0.1 to 4–116 35% and BI >4–10, (3%.

Chi square testing was done on the clinical features of patients, between each arm, and no differences found indicating that similar patients were recruited to the study).

### Outcomes

**Fig 1:**
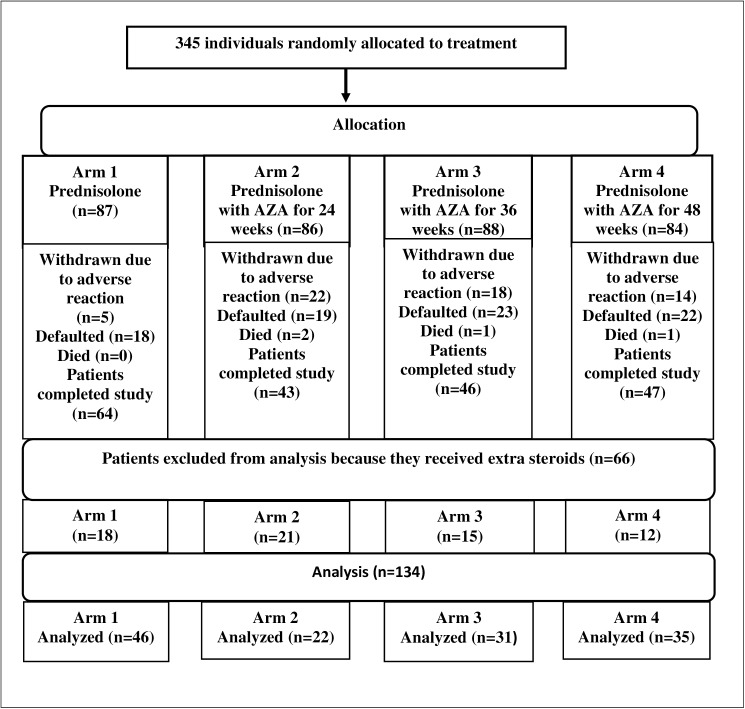
Azathioprine study Flow chart.

Two hundred patients completed the study. Fig 1 Azathioprine study Flow chart shows the outcome (completed, withdrawn, defaulted and died) for patients in each treatment arm. Default (20–26%) and withdrawal rates (5.7% to 25.5%) were lowest (20%) in the prednisolone only arm.

### Adverse events

**Table 2** shows that Cushingoid features (43%) and infections (35%) were the commonest events occurring at comparable rates across the four arms. Gastrointestinal symptoms and anaemia were more common, but not significantly, in patients treated with azathioprine, and four patients on azathioprine developed psychosis. Fifty-two out of 345 patients were withdrawn due to adverse events, pregnancy and developing ENL. The withdrawal rates between the arms ranged from 9–24%. In patients developing adverse events study drugs were withheld and the patients were monitored more closely and managed according to the protocol.

**Table 2:** Adverse Reactions encountered in the study (n = 345).

S.No	Adverse events	Pred Group (Arm 1) n = 87	Aza Group (2, 3 & 4) n = 258	Total n = 345	p-value
1	Cushingoid features	46.0%	40.7%	42.02	0.388
2	Infections	34.5%	36.0%	35.65	0.792
3	Hb drop >2 gm%	29.9%	34.9%	33.6	0.394
4	GI symptoms	21.8%	31.8%	29.28	0.078
5	Weight loss > 5 kg	1.1%	5.4%	4.35	0.091
6	Nocturia, polyuria, polydipsia	6.9%	2.3%	3.48	0.569
7	Comorbities, DM2, HTN	2.3%	3.9%	3.48	0.487
8	Psychological side effects	0.0%	2.3%	1.74	-
9	Hepatic & renal function derangement	1.1%	0.4%	0.56	0.420

Statistical testing was done between the rate of adverse events in the prednisolone and APC treated groups.

Anaemia was the most common reason for withdrawal (Table 3), affecting 59.3% patients and a significant interaction was found between patients being on azathioprine and developing anaemia (proportion z-test, p < 0.05). Patients taking dapsone were also at higher risk of developing anaemia, presumably due to an interaction between these two drugs. Nausea, vomiting, gastritis, loss of weight and a general ill-health were common and nine patients were withdrawn. Four patients developed infections including multi-dermatomal herpes zoster (2), infective gastroenteritis and hepatitis. The cause of the gastroenteritis and jaundice was not identified.

**Table 3:** Adverse conditions resulting in withdrawal (n = 59).

S. No	Adverse events	Pred Group (Arm 1) n = 87*		Aza Group (2, 3 & 4) n = 258*		Total n = 345*		p-value
		No	%	No	%	No	%	
1	Anemia	1	1.1%	34	13.2%	35	10.1%	0.001
2	GI symptoms	1	1.1%	21	8.1%	22	6.4%	0.021
3	Cushingoid features	1	1.1%	20	7.8%	21	6.1%	0.024
4	Infections	1	1.1%	10	3.9%	11	3.2%	0.199
5	Weight loss > 5 kg	0	0.0%	10	3.9%	10	2.9%	-
6	Mood alterations, psychosis or other mental problems	0	0.0%	4	1.6%	4	1.2%	-
7	Comorbities—DM2 HTN	0	0.0%	3	1.2%	3	0.9%	-
8	Hepatic & renal function derangement	1	1.1%	1	0.4%	2	0.6%	0.456
9	Nocturia, polyuria, polydipsia	0	0.0%	1	0.4%	1	0.3%	-

* Total no of enrollment.

Statistical testing was done between the rates of adverse events in the prednisolone and the APC treated groups.

Four patients became pregnant during the study and the study drugs were stopped as soon as the pregnancy was diagnosed. The pregnancies were diagnosed at six weeks. Three women chose to have their pregnancies terminated and one delivered a healthy baby.

Three patients developed ENL during the study. The study drugs were stopped and the patients were managed separately for their new immune complication in the clinic. Two patients had hepatic and renal function abnormalities which resolved after per protocol management. One patient developed sub-acute intestinal obstruction associated with an undescended testis and had surgery and was withdrawn from the study.

Most patients were withdrawn during the 2nd (22%) and 3rd (20%) months and were taking azathioprine. Patients continued to be withdrawn over the next seven study months with no withdrawals in the 11th and 12th month.

Four patients died during the study and their details are summarized in Table 4. All four patients were taking azathioprine as well as steroids and MDT. For each patient who died we tried to establish the cause of death and to establish whether azathioprine, prednisolone was the likely cause of death.

**Table 4:** Clinical Features and Laboratory investigations of patients who died.

	PA 10	PA 92	SA 102	CA 01
**Arm of trial**	4	2	2	3
**Age/Sex**	24/M	55/M	16/F	50/M
**MDT doses**	2	6	10	7
**Duration in trial**	2 wks	24 wks	12 wks	3 wks
**Lab findings**	Enrollment–normal	Enrollment- RBS– 159 Triglycerides—235	Enrollment–WNL	Enrollment -WNL
			Sudden drop in hemoglobin with severe pancytopenia PCR +ve for TB Chest Xray–Hilarlympadenopathy	Admitted for cellulitis
	2Wks -Leucopenia			
		Subsequent months RBS-474,309,220,450,158		
**Differential diagnosis**	Viral Fever	Cardiovascular accident	1)Community acquired pneumonia with Pancytopenia	Underlying sepsis
	Enteric fever			
		R/F—fluctuating sugars (steroid induced) and dyslipidemia	2)Coexisting undiagnosed infection (TB, HIV, Typhoid dengue fever	
**Place of death**	Govt hospital	Govt hospital	Govt Hospital	TLM hospital
**Therapy**	Supportive	Supportive	Referred when condition worsened	Supportive
**Attributed to Azathioprine**	**NO**	**NO**	**YES**	**NO**

Patient 1 PA1: This patient developed a high fever with loose stools. The differential diagnoses included viral fever and typhoid fever. He had been in the trial for 15 days. It is unlikely that the trial drugs caused his death.

Patient 2 PA92: This patient had a cardio-vascular accident (CVA) (diagnosis on death certificate). He developed steroid induced diabetes and had dyslipidemia. He had been in the trial for 24 weeks. His steroid treatment had been stopped at 20 weeks, four weeks before his death. We attribute his death to his underlying co-morbidity exacerbated by the steroid treatment.

Patient 3 SA 102: The cause of death in this lady was not established. She developed a pancytopenia after being on trial drugs for 12 weeks. Her azathioprine and MDT were stopped. She also developed progressive anaemia, high fever, and cough and there was a possibility that she had TB. An unvalidated PCR test for *M*. *tuberculosis* DNA was positive and she had hilar lymphadenopathy on chest X-ray. She was not tested for HIV. She died in another hospital in Delhi of unidentified infection. Her death was probably related to her taking azathioprine.

Patient 4 CA 01: This man had a probable myocardial infarction (MI) and sepsis. He was on the study drugs for 22 days. He had pre-diabetes and developed sepsis after starting medication. This could have been due to the steroid treatment or the azathioprine. The death also occurred soon after starting trials drugs so it is unlikely to be due to azathioprine.

### Intention to treat analysis

The trial outcomes were assessed by comparing the baseline with end of study clinical scores for each patient in each treatment arm. An Intention to Treat Analysis was done for 279 of the 345 patients entered into the study (excluding 66 patients who received a second course of prednisolone). For patients who did not complete the study (n = 145) their last assessment was taken as their endpoint). The combined score change and then the individual score components (skin, sensory, motor) are presented here.

**Fig 2:**
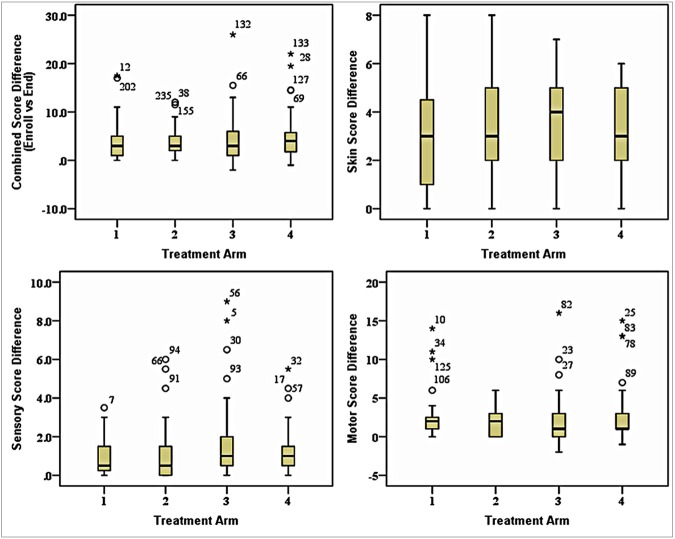
Comparison of the score (Skin, Sensory & Motor) differences for patients in each treatment arm from Baseline to Endpoint (n = 279) and for each parameter combined, skin, sensory, motor and combined scores.

Fig 2 shows the effect of treatmentin the 4 treatment arms for the 4 parameters, combined score, skin, sensory and motor scores differences for 279 patients. Three patients were worse and 65 patients had no or little change (0, 0.5, and 1). The median change in patients in arms 1, 2 and 3 was 3.0, and 4.0 for patients in arm 4. Sixteen (5.7%) patients improved substantially with score differences 10 and more. The baseline and endpoint score differences for patients in each arm were highly significant (p<0.001). However treatment with azathioprine did not improve outcomes, when the azathioprine treatment groups were compared with the steroids only treatment group, singly or combined. This is indicates that the improvement was due to the steroids and azathioprine did not enhance this improvement.

Fig 2 shows the skin score changes in 180 of the 279 patients who had Type 1 reaction (99 patients with only NFI were excluded). No patients deteriorated and 38 patients had no or little change (0 and 1). The median score changes for patients in arms 1, 2 and 4 were 3.0, and arm 3 was 4.0. Fifty-seven (31.6%) patients improved substantially, with score differences of 5 and more. The baseline and endpoint score differences for patients in arm 1, 2, 3 and 4 were highly significant (p<0.001), indicating that prednisolone treatment in these arms produced improvement and adding azathioprine did not improve skin outcomes in any arm.

Fig 2 shows the score differences for patients who had sensory loss (n = 104). No patients deteriorated and 69 (66.3%) patients had no or little change (0, 0.5 and 1). The median score change for patients in arm 1 and 2 was 0.5, and in arm 3 and 4 was 1.0. Seven (6.7%) patients improved substantially with a score difference of 5 and more. The score differences were significant for patients receiving 48 weeks azathioprine (p = 0.0002) and almost significant for patients in arms 1 and 3 (P values 0.0505 and 0.0512 respectively). This suggests that azathioprine might have a beneficial effect on sensory function.

Fig 2 shows the difference in motor scores for 152 patients who had motor loss. Three patients deteriorated and 72 patients had no or little change (0 and 1). The median change for patients in arms 1 and 2 was 2.0 and arms 3 and 4 was 1.0. Twenty (13.1%) patients improved substantially with score differences of 5 and more. Patients treated with prednisolone only and prednisolone plus 24 weeks of azathioprine had significant improvements in their score differences p 0.0002 and p 0.0001), no benefit was seen for treatment with 36 or 48 weeks of azathioprine with respect to motor function.

These figures also show that a few patients in the treatment groups responded to treatment, however their numbers were not significant.

### Analysis for patients who completed 20 weeks of treatment

A similar analysis was done on patients (n = 253) who completed 20 weeks of treatment and we found similar significant improvements in the combined and skin, sensory and motor scores but azathioprine did not enhance improvement.

### Recurrences

**Fig 3:**
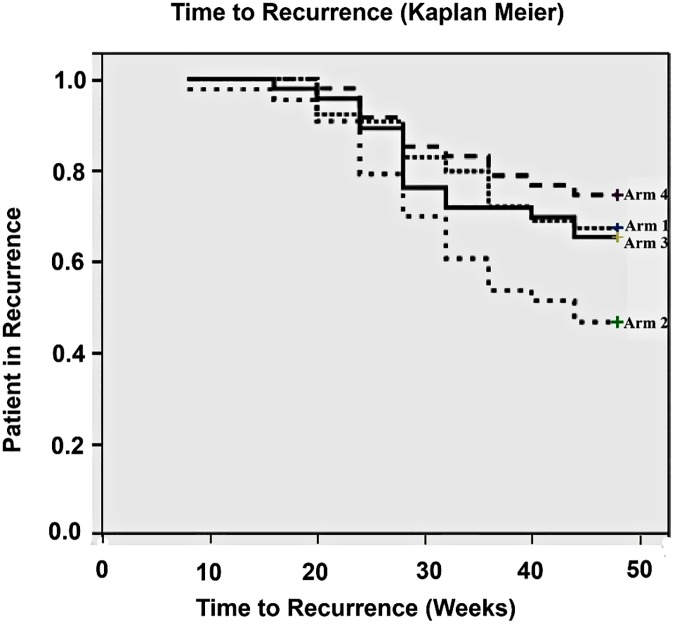
Kaplan Meier Curve showing Time to recurrence in each treatment arm.

Recurrence was calculated in the 200 patients who completed the study and 72 (36%) had recurrences. There were 21 (32.8%), 23 (53.5%), 16 (34.8%) and 12 (25.5%) recurrences in each arm respectively. The first recurrence occurred at 16 weeks and continued throughout the study. Sixty-six patients were given a further course of prednisolone (18, (28.0%), 21 (48.8%), 15 (32.6%) and 12 (25.5%), and no statistical differences were found between the need for extra prednisolone between patients in the different treatment arms. Fig 3 shows the time to recurrence in each treatment arm and no significant differences were found between the four arms, showing that treatment with azathioprine did not delay the time to recurrence, although patients with the 48-week azathioprine treatment had the lowest rate of recurrence.

### Per protocol analysis

We also did a per protocol analysis for patients who completed the treatment course (n = 134). The findings were very similar to the Intention to Treat Analysis (ITTA). The combined and sensory scores are very similar in this group at 20 weeks and 48 weeks. However there was a late improvement in motor score.

### Summaries of the analyses

The ITTA (n = 279) showed that there were significant baseline-end differences in scores for all the groups. Of the component scores, skin improved from baseline to end in all patients, sensory scores were only significantly improved in arm 4 and motor scores in arms 1 and 2. None of these differences were significantly different between the steroid and the steroid + azathioprine treatment arms. So prednisolone produces the benefit and azathioprine does not add to it. The small changes in treatments arms suggest that addition of azathioprine has a small effect in these patients.

**Fig 4:**
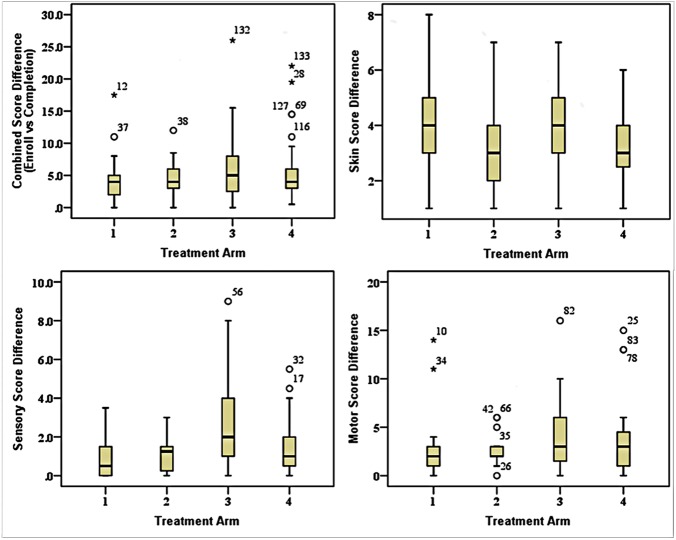
Comparison of the score (Skin, Sensory & Motor) differences for patients in each treatment arm between Baseline and 48 weeks in study (n = 134) and for each parameter combined, skin, sensory and motor.

Fig 4 shows the score differences for patients in each treatment arm and for the different parameters measured. The per protocol analysis show a similar picture with significant combined, skin and motor score changes from baseline to end for all treatment arms (Fig 4). Prednisolone treatment does not produce significant changes in sensory scores. This means that in this analysis for combined, skin and motor scores, prednisolone has an effect that azathioprine does not augment. For the sensory scores prednisolone is not effective. This also concurs with our finding that 67% of patients in the trial did not improve their sensory scores.

The 20 weeks analysis shows a significant benefit for the combined score and a significant benefit for azathioprine treatment. The skin scores in this group all improved, the sensory scores for arms 1, 2 and 4 and the motor improvement were all significant. The differences between azathioprine and prednisolone only treated patients were only significant for the 24 week azathioprine in the motor score.

These findings can be summarised as showing that prednisolone contributes the most to improvement and there is some evidence that azathioprine gives some improvement to the motor function but azathioprine does not improve sensory function.

### Nerve trunk function outcomes

Table 5 shows the nerve function outcomes at endpoint for all the peripheral nerve trunks by sensory and motor function. We compared the outcomes for each peripheral nerve trunk for patients receiving either prednisolone alone or APC. Each nerve was categorized into ‘unchanged’, ‘improved’ or ‘recovered’ separately for motor and sensory score differences at the end of the study No significant differences were found with the azathioprine treatment. The ulnar, posterior tibial and median nerves improved had the most improved sensory function and the median, lateral popliteal and ulnar nerves had the most improved motor function.

**Table 5:** New NFI- All affected nerve compared from baseline to endpoint of study assessing both motor and sensory function (n = 134).

Number affected Nerves (Both Rt& Lt)		Recovered	Improved	Unchanged	Worsened	Total
**Sensory Nerve**	Ulnar	31 (73.8%)	6 (14.3%)	5 (11.9%)	0 (0.0%)	42 (100%)
	Median	27 (64.3%)	4 (9.5%)	11 (26.2%)	0 (0.0%)	42 (100%)
	Posterior Tibial	18 (54.5%)	6 (18.2%)	8 (24.2%)	1 (3.0%)	33 (100%)
**Motor Nerve**	Facial	15 (35.7%)	18 (42.9%)	9 (21.4%)	0 (0.0%)	42 (100%)
	Ulnar	39 (52.0%)	21 (28.0%)	15 (20.0%)	0 (0.0%)	75 (100%)
	Median	26 (83.9%)	3 (9.7%)	2 (6.5%)	0 (0.0%)	31 (100%)
	Radial	3 (100.0%)	0 (0.0%)	0 (0.0%)	0 (0.0%)	3 (100%)
	Lateral Popliteal	21 (56.8%)	9 (24.3%)	7 (18.9%)	0 (0.0%)	37 (100%)

### Least and worst affected nerves

We hypothesized that the least affected nerve might show higher rates of recovery and the worst affected nerves the least improvement. These nerves were categorized by worst affected and least affected (definitions in Materials and Methods). Fifty-seven nerves were identified in the least affected category with recovery rates of 83% of nerves in the azathioprine + prednisolone treated groups and 78% in the prednisolone treated patients. Recovery occurred in all nerves with no nerve dominating. However there were only a few nerves in each category. In the worst affected nerve group a significant benefit for azathioprine treatment was found, with 82% worst nerves treated with Azathioprine plus steroids recovered and improved whereas only 66% worst affected nerves treated with steroids recovering (Z test p 0.0171).

### Old nerve function Loss

We also tested the hypothesis that old nerve damage would show less improvement, by looking at the score differences for nerves that had been classified as having old damage. The sensory scores of three patients deteriorated and 72 patients had no or little change (0, 0.5 and 1). The median change for patients in all four arms was 0.0. One out of 82 patients improved substantially with score difference 5 and more. Motor scores of five patients deteriorated and 39 patients had no or little change (0 and 1). Two patients improved by 5 and more. The median change for patients in arm 1, 3 and 4 was 0.0, and arm 2 was 1.5. Treatment with azathioprine did not affect old damage for either motor or sensory scores (comparison made between arm 1 and 2, 1 and 3, 1 and 4). This shows that most patients with old motor and/or sensory nerve damage do not improve.

**Fig 5:**
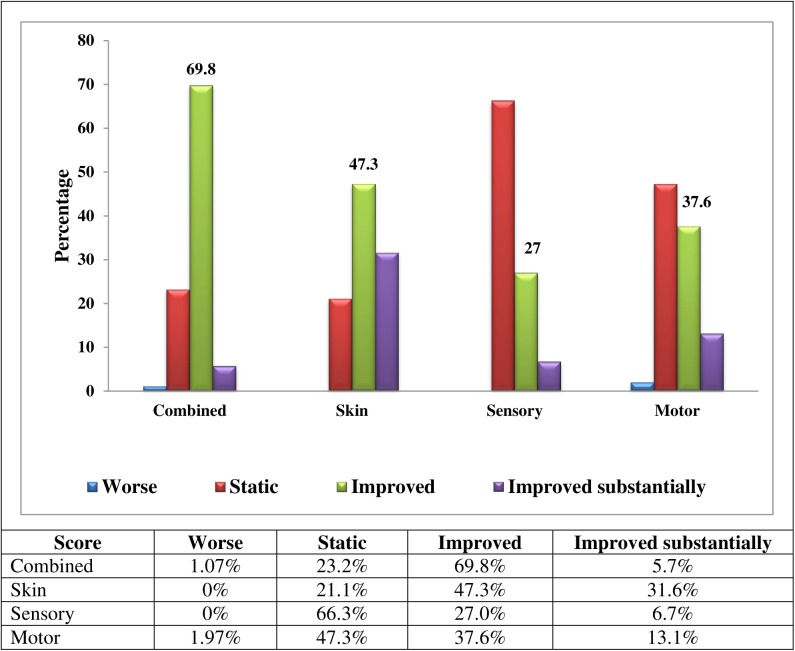
Score Changes by type of change and for each parameter (combined, skin, sensory and motor) for the whole cohort by the Intention to treat analysis (ITTA) (n = 279).

We analysed the score changes for all patients in the ITTA and found the following levels of change percentage wise for the combined treatment groups with the following categories, worse, static, improved and improved substantially. Combined (1.07, 23.2, 69.8, 5.7) Skin (0.0, 21.1, 47.3, 31.6) Sensory (0, 66.3, 27.0, 6.7) Motor (1.97, 47.3, 37.6, 13.1) Fig 5 shows the score changes for the whole cohort of the Intention to treat analysis by type of change and for each parameter combined, skin sensory and motor)

## Discussion

This was an important study because it is the first and largest RCT on a new treatment for leprosy T1R and nerve damage to be done with validated scores and validated tools for the skin and neurological outcome measures.[12] This trial was done to see if adding azathioprine to prednisolone could improve skin and nerve inflammation in leprosy reactions. A significant number of patients benefitted from the APC treatment with clinical score improvement but this was not significantly better than treating with P alone as analysed by both an Intention to Treat and per protocol analysis. We found that a 48 week course of azathioprine with prednisolone improves outcomes in sensory function, for motor outcomes we found that a 24 week course improved outcomes but was not superior to prednisolone alone. The effect was not seen consistently with the different lengths of treatment. The strengths and limitations of our study and the need for further studies will be discussed. Prevention of recurrence was our other outcome and 37% patients had a recurrence and so needed a further course of prednisolone.

Our study design tested three different lengths of treatment with azathioprine, this enabled us to test the effects of different durations of azathioprine, but we ended up with small numbers of patients in each treatment arm and even smaller numbers in each subgroup. Our main positive findings came from the analysis done of all patients who completed the twenty weeks of treatment. The effect of azathioprine is probably small; the previous study showed that azathioprine prednisolone combinations were only slightly better than prednisolone alone. So our study was probably under-powered to detect effects due to azathioprine. We might have under-detected benefits *vis a vis* sensory scores because we used only one monofilament the 2gm on the hand (2gm) and 10gm on the feet monofilament for sensory testing. Other studies have used the 2gm and 10gm on the hands and 10gm and 300gm on the feet.[6] The advantage of using two monofilaments or more is that one can detect a larger range of changes in sensory perception on the hands and feet. In our study we could not identify someone who could detect a 10gm but not a 2gm monofilament. We set a high threshold for sensory impairment. The poor improvement rate of 50% for motor improvements might be related to the difficulty of detecting small changes in muscle power. In the INFIR study a comparison of the ways of testing muscle power showed that manual testing was less sensitive than nerve conduction studies.[13] We used a fixed dose of 50mg azathioprine to facilitate the running of the trial. Our patient weights ranged from 32 – 95kg which translates into a weight adjusted dose of about 1mg/kg. This is a low dose but is used in patients with transplants and rheumatology conditions.

Although azathioprine was associated with adverse effects, the significant and serious ones were associated with steroid treatment. We found a significantly higher rate of anaemia in patients taking both azathioprine and dapsone. This is a novel finding and will be reported in detail elsewhere.

There were also four deaths, all in patients taking the prednisolone- azathiopirine combination and we were not able to identify a definitive cause of death for these patients. Two patients died within 22 days of starting azathioprine, so it is unlikely to have caused their deaths. However defining the cause of death was hampered by the absence of diagnostic and microbiological tests. Two patients may have had undiagnosed infections. Two of the hospitals (Champa and Purulia) are situated in remote areas without referral hospitals where complicated cases could be sent. Treatment with steroids is associated with development of new and worsening infections and diabetes. Screening for undiagnosed diabetes was part of the protocol and both patients had normal pre-trial glucose levels. So the steroid treatment might have caused the new diabetes since two patients had high peri-mortem glucose levels. The steroid part of the treatment regimen might have contributed to the cause of death in three patients, the fourth who developed pancytopenia was probably due to azathioprine.

It was surprising that azathioprine did not reduce the recurrence rate and the overall rate was 37% with no difference between the treatment arms, although the 48 week azathioprine course was associated with a lower recurrence rate (non-significant). In the INFIR study 30% of the study cohort developed a T1R or new nerve damage after the study started and in the methyl prednisolone study on the treatment of reactions 50% of the study participants had a recurrence. [14] [6] The recurrence rate of 36% highlights a major problem in the treatment of leprosy. [15]This phenomenon is linked to the persistent of inflammation in leprosy and strengthens the case for further studies on the immuno-pathology of leprosy and identifying the factors that contribute to continuing inflammation.

This study gives important trial data about the effect of steroids in leprosy reactions. These data have been collected in the trial setting and so are more robust than data collected from observational studies. These data show that the effect of steroids on skin inflammation is significant, with skin lesion inflammation improving significantly with 78.9% of patients showing some improvement. However both P and the APC were relatively ineffective at improving neurological outcomes; for sensory scores 66.3% patients were unchanged after 48 weeks and a further 33.7% had a change of only 1–0.5 points. The motor scores showed more improvement with 47% static and 50.7% improved. This replicates a Cochrane review which identified only three trials on steroids and nerve damage and found no benefit from steroid treatment.[4] Our data show that new nerve damage is most likely to improve, whilst patients with old nerve damage were unlikely to respond. This confirms previous findings by the Tripod 2 and 3 trials.[16, 17] These data are also important and novel in showing that is a significant adverse effects associated with steroid treatment. Forty five percent of patients had an adverse effect with Cushingoid features, infections and GI symptoms being common. These have not been reported in leprosy patients previously. Previous trials have not collected data on adverse effects systematically. In the methyl prednisolone study in Nepal data was collected and Cushingoid features were noted regularly. Observational studies will also miss collecting data on adverse effects and even deaths can be missed there. A recent study from Addis Ababa shows that when leprosy patients are treated with steroids rather than Thalidomide for ENL they have a mortality rate of 8%. This is further evidence on the importance of monitoring steroid use in leprosy settings carefully and being cautious. Osteoporosis is also a well-recognized adverse effect of steroid treatment. These patients were probably Vitamin D deficient and again highlight the possibility of unrecognized adverse effects.

Our data also show that it is difficult to switch off inflammation in the nerves, even with steroid treatment the improvement rates were very small. It was encouraging that few patients deteriorated once on steroid treatment. Previous studies which have looked at cytokine production in skin biopsies from patients with leprosy reactions have shown that inflammation can persist longer than 20 weeks.[18] Other studies have shown that cytokines have little effect on cytokine production in skin reactional lesion.[19] These studies and our data highlight the need for studies directed at understanding the mechanisms of inflammation in leprosy so that better tools for down regulating inflammation can be developed.

The strengths of this study are that the setting for treating leprosy patients was typical for patients in India, so our findings are applicable to other centres. We also had a very dedicated team who spent a lot of time following up patients and encouraging those with adverse effects.

Several research needs have been highlighted by this study. Trials are needed to identify the optimum duration of steroid treatment; this might vary for skin and nerve reactions. [21] Skin reactions might be successfully treated with a shorter course of steroids such as 12 weeks long. The Tenlep study is comparing 20 versus 32 weeks of treatment with steroids and this will guide future studies. Future work also needs to focus on trying to identify patients who do not respond to steroid treatment so that their treatment can be stopped or not implemented. Molecular markers might be one useful approach. A transcriptome approach identified several novel genes in leprosy reactions. [20]This could be developed. The 35% of patients who have a relapse are also a challenge and other trials might focus on the possible benefit of giving azathioprine to these patients and determining whether they could then be given a lower dose of steroids.

Other immune-suppressants have not been used systematically in the treatment of Type 1 leprosy reactions. We suggest that further small studies be done comparing prednisolone versus azathioprine plus prednisolone, given at a higher dose and for 48 weeks. More sensitive tools for measuring nerve function should be used, including a range of monofilaments for sensory testing and perhaps nerve conduction studies for assessing motor function.

When using azathioprine in leprosy patients on MDT we recommend that a non dapsone containing regimen is used to minimize the development of anaemia.

The service implications of our findings are that we should highlight awareness of the adverse effects of steroids when used in the field. We should also work to promote early detection of leprosy so that patients present before they have nerve damage. Future work should develop algorithms for identifying patients who do not respond to steroids and stopping their treatments rather than giving them long useless treatment courses.

In conclusion we have shown that it is difficult to improve on steroid treatment for leprosy inflammation. New approaches are needed to identify the underlying mechanisms and to develop new treatments.

## Supporting information

S1 Checklist STROBE Checklist. (DOCX)

S1 Protocol Study protocol. (DOC)
