# Supplementary material for: AZALEP a randomized controlled trial of azathioprine to treat leprosy nerve damage and Type 1 reactions in India: Main findings
Source: PLoS Negl Trop Dis. 2017 Mar 30;11(3):e0005348. doi: 10.1371/journal.pntd.0005348 (PMC5373510; doi:10.1371/journal.pntd.0005348)
Supplement: S1 Protocol — (DOC) [file pntd.0005348.s002.doc]

# Azathioprine Study: a randomized controlled trial of azathioprine in the management of acute neuritis and recurrent reactional skin lesions in leprosy.

## Study Protocol

Diana Lockwood ^1^

PSS Sundar Rao ^2^

Selvasekar Abraham^2^

Ruchika Chandna^2^

Prabu^2^

Address: ^1^ Department of Infectious and Tropical Diseases, London School of Hygiene

& Tropical Medicine, London, United Kingdom.

^2^ Research Resource Centre, the Leprosy Mission Trust India, Noida,

India.

#### Table of contents

[Background.…………………………………………………………………………….....3](#__RefHeading___Toc139355552)

[Introduction: 5](#__RefHeading___Toc139355554)

[Study Aims 5](#__RefHeading___Toc139355555)

[Study design and setting 6](#__RefHeading___Toc139355556)

[Identification of eligible patients 7](#__RefHeading___Toc139355557)

Determining the eligibility for the study…………………………………………………..7

Flow Chart for recruitment……………………………………………………..……………...10

[Randomization 10](#__RefHeading___Toc139355558)

[Enrollment 11](#__RefHeading___Toc139355560)

[Study interventions 12](#__RefHeading___Toc139355561)

[Patient follow-up procedure 12](#__RefHeading___Toc139355562)

[Patient Follow up : Management of Complications 14](#__RefHeading___Toc139355563)

[Patient outcome measures 15](#__RefHeading___Toc139355564)

[Primary outcomes 15](#__RefHeading___Toc139355565)

[Secondary outcomes 16](#__RefHeading___Toc139355566)

[Sample size 16](#__RefHeading___Toc139355567)

[Statistical analysis 16](#__RefHeading___Toc139355568)

[Time Plan for the Aza study 16](#__RefHeading___Toc139355569)

[Collection Of Data 17](#__RefHeading___Toc139355570)

[Competing interests 17](#__RefHeading___Toc139355571)

[Contribution of Authors 17](#__RefHeading___Toc139355572)

[Field Organization 17](#__RefHeading___Toc139355573)

[Acknowledgement 17](#__RefHeading___Toc139355574)

[Definitions 18](#__RefHeading___Toc139355575)

[References 19](#__RefHeading___Toc139355577)

## Background:

Leprosy is a chronic infectious disease of the skin and nerves caused by the intracellular pathogen *Mycobacterium leprae*. It is characterized by a spectrum of clinical forms depending on the host's immune response to *M. leprae*. Leprosy may be complicated by inflammatory reactions. Type 1 (reversal) reactions (T1R), common in borderline forms of leprosy, are due to delayed-type hypersensitivity and an increase in CMI to *M. leprae* antigens.

Preventing peripheral nerve damage is a major challenge in leprosy. Nerve damage occurs commonly in Type 1 reaction (T1R), and may be irreversible if not treated early and adequately^1 2 3^. Acute neuritis may also occur without the skin lesions of Type1 reactions.^4^

Type1Reactions are characterized by acute granulomatous inflammation that can produce destruction of nerves even to the extent of causing caseous necrosis of the nerve tissue and irreversible paralysis. The swelling of the nerves due to sudden increase in inflammatory cells and oedema within an unyielding perineurium produce ischaemia and transient paralysis^5^.

Corticosteroids are the accepted method of treating nerve function impairment and reactions in leprosy^6^. The effectiveness of steroids compared to placebo is uncertain.^7^ Corticosteroids have been used since 1950 for treating reversal reactions^8 9^. They are used because the TIR skin and nerve lesion show inflammation. Type 1 reactions, are due to delayed-type hypersensitivity and an increase in CMI to *M. leprae* antigens. A transient increase in the CMI response to antigens of *M.leprae* has also been reported lasting for weeks and then declining^10^. Prednisolone both decreases inflammation and suppresses the CMI.

Steroid courses used in the 1950s and 1960s were usually short and low dose, although by the 1980s longer courses and higher doses were being recommended.[[1]](#endnote-2)

Currently, standardized regimens, e.g. Prednipac (packaged course of steroids starting at 40 mg per day and reducing over 12 weeks), are used under field conditions and for out patients as they have been found to be relatively safer. ^12^ However a recent multi centre, double blind, randomized controlled trial showed a clear superiority of longer duration steroids for 20 weeks compared to a 12-week regimen^17^. This study will incorporate the current evidence on duration of treatment and use a course of prednisolone tapered over 20 weeks.

Treatment with corticosteroids improves both reactional skin lesions and neuritis. A retrospective study reported only a 50% improvement in clinical signs and symptoms of reactions involving nerves^13^, while other studies have reported improvement in nerve function of 60% to 70%.^14^ However upto 30% of patients with type 1 reactions or nerve function impairment may not improve with prednisolone.^15^

Patients treated with the standardized steroid course often require additional steroids while the dose is tapered – a recent trial conducted in Nepal showed 50% of patients treated with a 12 week prednisolone regimen required further steroids.^16^ The need for additional steroids prolongs the duration of steroid treatment and the total amount of steroid used. Another problem with using corticosteroids is the associated adverse effects.^8^ Concerns over such adverse effects of steroids used under field conditions have lead to pressure to keep the dose and duration to a minimum.^17^

The risks of adverse effects with standardized regimens of corticosteroids for the treatment of leprosy reaction in the field have been described.^18^

The adverse effects associated with corticosteroid therapy can be classified into minor such as moon face, fungal infections, acne and gastric pain. The major adverse effects being gastric ulcer, glaucoma, diabetes, hypertension, osteoporosis, cataract and a range of psychiatric disturbances ranging from mood alterations to psychosis. Besides these life-threatening infections can also occur. The risk of minor adverse effects while being on prednisolone was 16 % and 2% for major side effects. Dermatological side effects namely moon face, acne and fungal infections were seen in 6.5 % of patients on prednisolone .^19^

There is an urgent need for an adjunct to steroids in the treatment of type I reactions because:

1. 30-50% of patient with acute neuritis do not show neurological improvement when treated with the standard steroid regimens.
2. There is flare-up of skin lesions while steroid doses are tapered down.
3. Some patients require prolonged courses of steroids and become steroid-dependent.
4. Alternative treatments are needed for patients with contraindications to corticosteroids
5. Alternative treatments are needed for patients with severe neuritis who do not improve on steroid treatment.

Azathioprine is an immuno suppressant that has been used in other immune mediated diseases both as monotherapy and in combination with prednisolone as a steroid-sparing agent.^20^ It is metabolized to mercaptopurine by the enzyme thiopurine methyltransferase. It is widely used for organ transplant recipients and to treat a number of autoimmune conditions, usually when corticosteroid therapy alone provides inadequate control. ^21^

A recent trial conducted in Nepal using a three-month course of Azathioprine along with Prednisolone showed that addition of Azathioprine reduces the corticosteroid requirement for treatment of type 1 reactions. Although the clinical outcomes were similar for patients treated with either the prednisolone/azathioprine combination or prednisolone alone, the overall dose of prednisolone was less in those on the combined regimen.^16^ One of the shortcomings of the Nepal study was that the azathioprine was not used for a long enough duration.

Adverse effects due to azathioprine may occur in upto 15% of patients.^21^ The commonest adverse reactions that occur with azathioprine are nausea and vomiting and mucosal ulcers, occurring in 60% of patients.^22^ Azathioprine also causes significant bone marrow suppression (upto 5%) and can potentially cause leucopenia (3.8%), thrombocytopenia (2%) and even pancytopenia (0.4%).^23^ There may be an increased risk of infections and different malignancies. Pancreatitis, skin rashes and drug fever have been reported on rare occasions.^24^ Use of azathioprine in pregnancy has shown that the placenta may act as a barrier to its passage and current evidence of safety in pregnancy is reassuring.^25, 26^

## Introduction:

## Study Aims

The primary aim of this study is to compare the neurological outcomes in patients with type 1 reactions and recurrent type I reactions in skin lesions, when they are treated with either Azathioprine in addition to a 20-week course of prednisolone or prednisolone alone.

The objectives of the study are: -

1. Improves dermatological outcomes in Type 1 reactions.

2. Improves neurological outcomes in Type 1 reactions and neuritis

3. Reduces the recurrence rate (i.e. requirement for further treatment with steroids) in patients with T1R and neuritis.

4. To compare the effectiveness of different duration of treatment with Azathioprine in T1R and neuritis.

***Methods***

## Study design and setting

This study is a randomized double blind controlled clinical trial with continuous enrollment of individuals into 4 arms of the study.

The participating centres are:

- TLM hospital Faizabad, (UP)
- TLM Hospital Shahdara (Delhi).
- TLM Hospital Purulia (West Bengal)
- TLM Hospital, Champa

The study will compare Azathioprine when used with prednisolone against prednisolone used alone, for the treatment of acute neuritis and recurrent reactional skin lesions.

Azathioprine will be used at three durations of 24 weeks, 36 weeks and 48 weeks. All patients will complete 48 weeks of treatment comprising Prednisolone (20 weeks standard therapy) + Azathioprine/Placebo.

Clinical measurement of nerve function and skin appearance will be used to determine the outcomes.

##

## Identification of eligible patients

Eligible patients will be identified from among the patients undergoing regular skin examination, and nerve function assessment at the physiotherapy units in the four participating TLM hospitals.

The physiotherapy unit at each of the participating hospitals will flag all patients presenting with new nerve function impairment or recurrent reactional skin lesions, these flagged patients will be “fast tracked” and will taken to the designated medical officer for the study.

The patient would then be screened for eligibility; the pre-treatment assessment form will be filled in, if found eligible would be offered a choice of participating in the study subject to the patient consenting to participate in the study.

#### Determining the eligibility for the study

All patients with a diagnosis of leprosy (whether paucibacillary or multibacillary) and evidence of recent onset neuritis or recurrent skin lesions in type 1 reactions will be included in the study as long as he/she satisfies the inclusion criteria and does not have any absolute exclusion criteria listed in table 1. For the purpose of this study neuritis will be defined as: -

- Spontaneous nerve pain, paraesthesia or tenderness (or)
- New sensory or motor impairment of recent onset detected by voluntary muscle testing and sensory testing (or)
- Mixed sensory and motor signs.
  - The MDT status (past or current) of the patient does not affect eligibility provided they have recent neuritis or recurrent reactional skin lesions.
- Patients who are already on steroids (started within the past one month) will be included if they present with new nerve damage or fresh evidence of neuritis while on treatment with steroids.

The local MO decides whether the patient can be included in the study.

#### Inclusion Criteria:

Patients with acute (< 6 months) neuritis with or without accompanying type 1 reaction or recurrent reactional skin lesions without neuritis.

#### Exclusion criteria

- Age less than 15 yrs
- Weight less than 30 kgs
- Confirmed Pregnancy (based on the LMP + Pregcolor strip test)
- Patient on treatment for Tuberculosis.
- Patients with known HIV sero-positivity.
- Patients with Hepatic dysfunction as evidenced by recent history of jaundice and abnormal LFTs on laboratory testing.
- Patients with pre-existing bone marrow dysfunction as evidenced by, abnormal counts, spleenomegaly or on examination.
- Patients with Hypertension (Sustained Diastolic Blood Pressure greater than 90 mm Hg) and Diabetes (Fasting Blood Sugar greater than 110mg/dl or random blood sugar greater than 200 mg/d) are not to be immediately included in the study. However once the condition is brought under control by appropriate medications, they can be included in the study provided they fulfil other criteria.
- Patients unable to comply with monitoring and follow up requirements.
- Patients presenting with Type 2 reactions.

#### Exclusion criteria

- Age less than 15 yrs
- Weight less than 30 kgs

.

#### TABLE 1

#### Exclusion criteria

- Age less than 15 yrs
- Weight less than 30 kgs
- Confirmed Pregnancy (based on the LMP + Pregcolor strip test)
- Patient on treatment for Tuberculosis.
- Patients with known HIV sero-positivity.
- Patients with Hepatic dysfunction as evidenced by recent history of jaundice and abnormal LFTs on laboratory testing.
- Patients with pre-existing bone marrow dysfunction as evidenced by, abnormal counts, spleenomegaly or on examination.
- Patients with Hypertension (Sustained Diastolic Blood Pressure greater than 90 mm Hg) and Diabetes (Fasting Blood Sugar greater than 110mg/dl or random blood sugar greater than 200 mg/d) are not to be immediately included in the study. However once the condition is brought under control by appropriate medications, they can be included in the study provided they fulfil other criteria.
- Patients unable to comply with monitoring and follow up requirements.
- Patients presenting with Type 2 reactions.

All patients found eligible will be invited to participate in the study. They will receive a detailed information sheet about the Aza study (in the local language); a patient counselor will help them understand the details of the study. They will be advised to return to the study MO after having fully understood these details; they will then give signed informed consent to participate in the study in the presence of the study MO, and will finally be included.

Patients who are either ineligible for trial entry or who do not wish to participate are treated as per the routine practice in that hospital.

***Flow Chart for Recruitment***

NFI

Recurrent reactional skin lesions

Informed

Consent

(Form 1)

No

yes

Full Blood Count

Exclude

Hb < 7 gm

TLC < 3000

Platelets < 1 lac

No

Register for trial

(Form 2)

Randomize

Arm 1

Arm 2

Arm 3

Arm 4

Yes

Age < 15 yrs

Wt < 30kgs

Pregnancy

On treatment for TB

H/O Jaundice in last 3 m

Pre-existing bone marrow disease

Hepatic Dysfunction

Known HIV positive

Unable to come regularly

Form E

Yes

Yes

No

NFI < 6 months

Or recurrent reactional skin lesions

Form 3.0

Baseline history

& Examination

Lab tests

Randomised Study Patient

Form 4.0

Baseline ST

Form 5.0

Baseline VMT

Form 6.0

Baseline Lab Investigations

##

Allot 4 weeks treatment box

Follow up Visit

Lab tests

Form 6.1

Clinical

Form 3.1

ST

Form 4.1

VMT

Form 5.1

Serious

Adverse effects

Hb < 7.0

TLC < 3000

Platelet

Count < 1 lakh

Break

Code

Worsening

Improvement

Add steroids

Continue in study

Normal

SGOT < 50

Creatinine < 2.0

Treat accordingly

## Randomization

At the Randomization clinic the MO once again checks to ensure that the patient satisfies both inclusion and absolute exclusion criteria. S/he will fill in the Aza study Registration form (**Form 2**).

If a patient (and parent/guardian where age is less than 18 years) is willing to take part, the MO then obtains an informed written consent **(Form 1)** from the patient (parent/guardian) prior to randomization.

Block randomization will be used to ensure equal recruitment into all the four arms.

The randomization schedule will be developed by an independent unit and the random allocation number will be provided to the medical superintendent at each of the hospitals.

## Enrollment

At enrollment of a patient the MO will collect a randomization number and its corresponding packet that contains the relevant treatment arm for the entire length of the study. The MO will do a detailed clinical examination and fill in **FORM 3.0**

The randomization number will be noted on all the Aza study forms, the patient identity card and the patient medical record, the MO will then arrange for the appropriate lab investigations including the baseline punch biopsy. The MO will fill in the Lab results in **FORM 6.0** and sign the same before issuing the first two weeks blister pack to the patient.

The MO will explain in detail all the precautions (as listed in page 35 of the field manual) to be taken while on treatment in the study, and impress upon the patient the need for regular follow up.

## Study interventions

A standardised course of prednisolone starting at 40mg per day and tapered over 2 weekly reducing doses is the accepted practice for treatment of Type 1 reaction under field conditions and in outpatients.^4^

There are four arms to the study, three interventional arms and one control arm. The four arms of the study are depicted in table 2.

**TABLE 2 :**

|  | 1 | 2 | 3 | 4 | 5 | 6 | 7 | 8 | 9 | 10 | 11 | 12 |
| --- | --- | --- | --- | --- | --- | --- | --- | --- | --- | --- | --- | --- |
|  | Wk  4 | Wk  8 | Wk 12 | Wk 16 | Wk 20 | Wk 24 | Wk 28 | Wk 32 | Wk 36 | Wk 40 | Wk 44 | Wk 48 |
|  |  |  |  |  |  |  |  |  |  |  |  |  |
| Arm 1 | Prednisolone | |  |  |  |  |  |  |  |  |  |  |
|  |  |  |  |  |  |  |  |  |  |  |  |  |
|  | Placebo | | | | | | | | | | | |
| Arm 2 | Prednisolone | |  |  |  |  | | | | | | |
|  | Azathioprine | | | | | |  | | | | | |
|  |  |  |  |  |  |  | Placebo | | | | | |
|  | | | | | | | | | | | | |
| Arm 3 | Prednisolone | |  |  |  |  | | | | | | |
|  | Azathioprine | | | | | | | | |  | | |
|  |  |  |  |  |  |  |  |  |  | Placebo | | |
|  | | | | | | | | | | | | |
| Arm 4 | Prednisolone | |  |  |  |  | | | | | | |
|  | Azathioprine | | | | | | | | | | | |

## Patient follow-up procedure

Patients will be followed up from trial entry until the end of the study. Follow up is composed of several elements. A follow up schedule is summarized in the flow chart.

1. At baseline the patient will have laboratory investigations. This will comprise Smear Bacteriological and Morphological Indices (Smear BI and MI); a complete blood count (Hb%, Total Leucocyte Count and Total Platelet Count); Serum Creatinine; Serum AST and ALT.
2. Patients will be followed up fortnightly for the first 8 weeks and then at 4 week intervals with a full blood count and liver function tests done at each visit
3. At each follow up visit a physiotherapist will review the patient nerve function (voluntary muscle test (VMT) and sensory testing (ST) assessment and record the details in specially designed forms. in **FORM 4.1 (**each subsequent visit is indicated as **4.2 to 4.14**) and **FORM** **5.1** to **5.14** (corresponding to the visit number).
4. The PI / study MO will review the patient during follow up visit and evaluate for improvement / worsening of the neuritis. They will record evidence of any primary or secondary end points and compliance with the medication, specifically looking for adverse effects of the drugs. This will be recorded in **FORMs 3.1** to **3.14.**
5. Any participant who has not returned back for a follow up for more than 2 weeks will be contacted at his / her home by phone or personal visit. And attempt will be made to bring them back to the hospital for assessment. A patient who returns to the clinic within 4 weeks of the last visit will be continued in the study and those who do not return within 4 weeks will be removed as dropped out from the study
6. After 20 weeks if a patient wishes to go out of station he/she maybe given 8 weeks of treatment at one point of time and a single follow-up session maybe missed. If two follow-ups are missed concurrently the patient will be treated as per routine clinical practices but will have to be removed from the study.
7. Any patient who has missed their treatment for 4 weeks concurrently will be removed from the study and will be treated as per routine clinical practices.
8. The records of the patients will be flagged in the TLM Patient information systems, to ensure that the study team is informed of any deaths that occur at home. The MO should visit the family to establish the cause of death

## Patient Follow up: Management of Complications

1. If the neuritis / type 1 reaction worsens (as per defined criteria of VMT and sensory testing) while the steroid is being tapered, then the dose of Prednisolone will be STEPPED UP to the preceding dose. After 20 weeks when Prednisolone has been stopped, if worsening occurs, the patient will be restarted at 40 mg of Prednisolone.
2. If the patient develops medical complications like leucopenia, jaundice or anemia it is imperative that this is investigated as per the protocol to determine the etiology. It should not be assumed that complications are caused by the trial drugs.
3. In case the patient is detected to have potential adverse effects of the drug(s) by lab investigations as listed in the flow diagram, then code will be broken by study coordinator
4. In case the patient develops clinical symptoms and signs suggestive of a severe adverse effect, such as systemic sepsis or gastro intestinal bleed then the patient will be admitted into the corresponding hospital and treated accordingly. The Study Coordinator will be contacted and the case reviewed. The Study coordinator will determine whether to break the code or continue the trial after symptomatic treatment.
5. In case a patient gets pregnant while enrolled in the study, she can be reassured that Azathioprine is highly unlikely to affect the fetus and continued in the study. However she may discontinue if she so wishes, in which case her nerve function assessments will be continued as per schedule.
6. In case of infections such as Tuberculosis, patient will be started on Anti Tuberculosis Treatment (ATT) after relevant investigations and continued in the study.

## Criteria for breaking the code

| Laboratory Parameter | Cut-off Point |
| --- | --- |
| Hb | < 7 g % |
| TLC | < 3000 |
| Platelet Count | < 1 lakhs |
| SGOT | > 50 |
| Creatinine | > 2 |

In case of abnormal blood counts, the following guidelines should be followed:

| Laboratory Parameter | Level | Action |
| --- | --- | --- |
| Total WBC | < 3 X 10^9^/l | Break Code / Continue steroids |
| Absolute Neutrophil Count | < 1.0 X 10^9^ /l | Break Code / Continue steroids |
| Absolute Neutrophil Count | < 0.5 X 10^9^ /l | **If febrile**, start **antibiotic** and look for source of infection  As per guidelines in Field Manual |
| Hb | < 7.0 g/dl | Break Code / Continue steroids |
| Hb | Fall > 3 g/dl from baseline | **Break Code / Continue steroids** |
| Platelets | < 100000 | **Break Code/ Continue steroids** |
| Serum Creatinine | > 50 % above baseline | **Break Code / Continue steroids** |
| SGOT | > 2 X upper limit of normal | **Break Code / Continue steroids** |

## Patient outcome measures

### Primary outcomes

1. **Recovery of nerve function**

- Absolute improvement in nerve function as measured by Voluntary Muscle testing (one point improvement in VMT score) and Sensory testing (two point improvement in ST score) using standard methods as defined in the field manual.

1. **Recurrence of neuritis during or after treatment**

- Recurrence of neuritis is defined as a new nerve function impairment presenting in the form of a decrease in VMT score by one point and an ST score by more than 2 points either during the study period or during the follow up period and present for two consecutive testings.

### Secondary outcomes

1. Resolution of skin lesions
2. Recurrence of reaction manifest as skin reactions.

## Sample size

The sample size calculation was based on the expected improvements in the combined scores (skin, sensory & motor) that the addition of azathioprine to prednisolone would produce. It was calculated that the addition of azathioprine would produce a 25% improvement in the combined scores. This would be a score of 4 allowing for an alpha of 0.05 and beta of 0.80, the minimum sample size is calculated as follows:

n=2x[(Zα +Z1-β)xSD)/ d]2 =2x[(1.96+0.842))x2)/1]2 = 63 per group.

We assumed a ‘lost to follow up’ rate of 15%, and dropout rate due to adverse reactions of 10 %,so minimum of 78 patients would be required per arm, and a total of 312.

## Statistical analysis

The primary analysis will be a comparison of azathioprine plus prednisolone with prednisolone alone for achieving the primary outcome following the “intention to treat” principle. Intention to treat analysis will also be performed for the secondary outcomes.

Analysis of endpoints, comparing time to event in the four arms will be performed.

Pre-planned sub group analysis will be performed for the following subgroups (male, female; MB, PB; previous T1R, new T1R).

A secondary on treatment analysis will be performed to explore the requirement of additional steroids as well as adverse effects with these drugs.

## Time Plan for the Aza study

The training of the Investigators and the physiotherapists will begin in July 2007, recruitment of participants will begin from August 2007 and is planned to continue until the required numbers are enrolled.

## Collection of Data

Data collection and entry: A computerized database will be used for collection of the patient clinical data a paper copy of the Aza study forms will be maintained with the MO at each participating hospital. The data will be send in an encrypted format on a weekly basis to the study coordinating centre at RRC from the participating hospitals by email, which will be made centrally monitored at RRC and LSHTM. The data and clinical safety monitoring committee will receive updates on the data every six months.

## Competing interests

None of the authors have any competing interests arising from this research.

## Contribution of Authors

Diana Lockwood was responsible for identifying the research question and revising the protocol. Oommen John was responsible for the drafting of the study protocol and study forms. Sundar Rao was responsible for the sample size and statistical inputs**.**

## Field Organization

The Study will be coordinated at TLM RRC by Dr Selvasekar Abraham and Dr Ruchika Mehndiratta. Dr. N.K.Nanda and Dr J. Palla, will represent the TLM centres. Dr. Diana Lockwood would coordinate the study from LSHTM.

Dr PSS Sundar Rao will coordinate the randomization procedure and statistical analysis, and will oversee the data management.

### *Acknowledgements*

The Aza study group would like to thank The Moulton Charitable trust for funding this study and International Research Committee and South Asia Research committee of TLM for their comments on the study and permission for the study to be conducted in TLM hospitals.

### *Definitions:*

### *Neuritis*: A leprosy patient is diagnosed as having neuritis when he/she has any of the following:

- Spontaneous nerve pain, paraesthesia or tenderness (or)
- New sensory or motor impairment of recent onset detected by voluntary muscle testing and sensory testing (or)
- Mixed sensory and motor signs

*Acute Neuritis*: For neuritis to be acute it has to have appeared within the last six months (defined either by previous testing or patients history of new sensory or motor loss)

*Type1Reaction* (T1R) is defined as the development of an acute or subacute inflammation affecting the skin and / or nerves occurring in leprosy, due to a hypersensitivity to *M.leprae* antigens, resulting in a change in the appearance of skin lesions and / or nerve damage and disability.

A T1R is diagnosed when a patient has erythema and edema of skin lesions. There may be accompanying neuritis and edema of the hands, feet and face. The skin signs are obligatory; the nerve and general signs optional.

*Acute T1R* is defined as the presence of erythematous and raised skin lesions with or without evidence of neuritis of less than 6 months duration prior to presentation.

*Recurrent reactional skin lesions* are defined as newly erythematous lesions that have either developed off steroids or whilst on steroids.

*Silent Neuropathy (SN)*

A patient is defined as having silent neuropathy when he/she has sensory and/or motor impairment of recent onset (< 6 months duration) in an area innervated by one or more nerve without signs of a reaction (RR or ENL) or nerve pain and with or without tenderness.

*Sensory Impairment:* A patient is diagnosed as having sensory impairment in any of the following situations: the monofilament threshold was increased from the normal threshold (2gm/purple for the hand and 10gm/orange for the foot) by three or more levels (filaments) on any site, OR two levels on one site AND at least one level on another site, or one level on three or more sites for one nerve.

*Motor Impairment:* A patient will be diagnosed as having motor impairment if the VMT score for any muscle is four or less on the 0-5 (modified) MRC scale.

*Worsening of skin lesions:* is defined as occurrence of a fresh episode of acute skin inflammation with or without edema while enrolled in the study.

*Worsening of Acute Neuritis*: is defined as evidence of nerve function impairment as evidenced by at least a single point decrease in VMT score or a two-point decrease in ST score from baseline, in two consecutive testings.

### References:

1. ^1^ Pearson JM, Ross WF. Involvement in leprosy--pathology, differential diagnosis and principles of management. Lepr Rev. 1975;46:199-212.

   ^2^ Bjune G. Reactions in leprosy. Lepr Rev. 1983 :61-7.

   ^3^ Croft RP, Nicholls PG, Richardus JH, Smith WCS. The treatment of acute nerve function impairment in leprosy: results from a prospective cohort study in Bangladesh. Lepr. Rev.2000; **71:**154-168.

   ^4^ WHO Expert Committee on Leprosy. World Health Organ Tech Rep Series. 1998;874:1-43.

   ^5^ Job CK. Nerve in reversal reaction. Indian J Lepr.1996;68:43-7.

   ^6^ Van Brakel WH, Khawas IB. Nerve function impairment in leprosy: an epidemiological and clinical study--Part 2: Results of steroid treatment. Lepr Rev. 1996;67:104-118.

   ^7^ Lockwood D, Vinayakumar NS, Stanley JN, McAdam KP, Colston MJ. Clinical features and outcome of reversal (type 1) reactions in Hyderabad, India. Int. J. Lepr. Other Mycobact. Dis.1993; **61:**8-15.

   ^8^ Lowe J. A.C.T.H.and cortisone in treatment of complications of leprosy. Br Med J. 1952 ;2:746-9.

   ^9^ Cochrane RJ, Jopling WH. The place of cortisone and corticotrophin in the treatment of certain acute phases of leprosy. Lepr Rev. 1957;28:5-10.

   ^10^ Manandhar R, Shrestha N, Butlin CR, Roche PW. High levels of inflammatory cytokines are associated with poor clinical response to steroid treatment and recurrent episodes of type 1 reactions in leprosy. Clin. Exp. Immunol. 2002;128:333-338.

   ^11^ Pearson JM. The use of corticosteroids in leprosy. Lepr Rev. 1981;52:293-8.

   ^12^ WHO Expert Committee on Leprosy. World Health Organ Tech Rep Series. 1998;874:22-23.

   ^13^ Lockwood D, Vinayakumar NS, Stanley JN, McAdam KP, Colston MJ. Clinical features and outcome of reversal (type 1) reactions in Hyderabad, India. Int. J. Lepr. Other Mycobact. Dis.1993; **61:**8-15.

   ^14^ Sugumaran DST. Steroid therapy for paralytic deformities in Leprosy. Int J Lepr,1997;65: 337-344.

   ^15^ Bernick 1997

   ^16^ Marlowe SN,Hawsworth RA,Butlin CR, Nicholls PG, Lockwood D. Clinical outcomes in a randomized controlled study comparing azathioprine and prednisolone versus prednisolone alone in the treatment of severe leprosy type 1 reactions in Nepal. Trans R Soc Trop Med Hyg. 2004 Oct;98(10):602-9. 2004

   ^17^ Rao PSSS, Sugumaran DST, Richard J, Smith WCS. Multi-centre, double blind randomized trial of three steroid regimens in the treatment of type 1 reactions in leprosy. Lepr Rev, 2006; 77: 25 – 33.

   ^18^ Richardus JH, Smith CS. The risk of standardized regimens of corticosteroids for the treatment of leprosy reactions in the field. Lepr Rev. 1995;66:328-9.

   ^19^ Richardus JH, Withington SG, Anderson AM, Croft RP, Nicholls PG, Van Brakel WH, Smith W. Adverse events of standardized regimens of corticosteroids for prophylaxis and treatment of nerve function impairment in leprosy: results from the 'TRIPOD' trials. Lepr Rev. 2003; 74:319-327.

   ^20^ Pearson DC,May GR, Fick GH. Azathioprine and 6-mercaptopurine in Crohn disease. A meta-analysis.
   Ann Intern Med. 1995 Jul 15;123(2):132-42.

   ^21^ Lamers CB. Azathioprine: an update on clinical efficacy and safety in inflammatory bowel disease.
   Scand J Gastroenterol Suppl. 1999;230:111-5.

   ^22^ Drugs and Therapeutics bulletin, 1994.

   ^23^ Connell WR, Kamm MA, Ritchie JK, Lennard-Jones JE. Bone marrow toxicity caused by azathioprine in inflammatory bowel disease: 27 years of experience. Gut. 1993 Aug; 34(8):1081-5.

   ^24^ Callen JP, Spencer LV, Burruss JB, Holtman J. Azathioprine. An effective, corticosteroid-sparing therapy for patients with recalcitrant cutaneous lupus erythematosus or with recalcitrant cutaneous leukocytoclastic vasculitis. Arch Dermatol. 1991 Apr;127(4):515-22.

   ^25^ de Boer NK, [Van Elburg RM](http://www.ncbi.nlm.nih.gov/entrez/query.fcgi?db=pubmed&cmd=Search&itool=pubmed_Abstract&term="Van+Elburg+RM"%5BAuthor%5D), [Wilhelm AJ](http://www.ncbi.nlm.nih.gov/entrez/query.fcgi?db=pubmed&cmd=Search&itool=pubmed_Abstract&term="Wilhelm+AJ"%5BAuthor%5D), [Remmink AJ](http://www.ncbi.nlm.nih.gov/entrez/query.fcgi?db=pubmed&cmd=Search&itool=pubmed_Abstract&term="Remmink+AJ"%5BAuthor%5D), [Van Vugt JM](http://www.ncbi.nlm.nih.gov/entrez/query.fcgi?db=pubmed&cmd=Search&itool=pubmed_Abstract&term="Van+Vugt+JM"%5BAuthor%5D), [Mulder CJ](http://www.ncbi.nlm.nih.gov/entrez/query.fcgi?db=pubmed&cmd=Search&itool=pubmed_Abstract&term="Mulder+CJ"%5BAuthor%5D), [Van Bodegraven AA](http://www.ncbi.nlm.nih.gov/entrez/query.fcgi?db=pubmed&cmd=Search&itool=pubmed_Abstract&term="Van+Bodegraven+AA"%5BAuthor%5D)6-Thioguanine for Crohn's disease during pregnancy: thiopurine metabolite measurements in both mother and child. [Scand J Gastroenterol.](javascript:AL_get(this, 'jour', 'Scand J Gastroenterol.');) 2005 Nov;40(11):1374-7.

   ^26^ [de Boer NK](http://www.ncbi.nlm.nih.gov/entrez/query.fcgi?db=pubmed&cmd=Search&itool=pubmed_Abstract&term="de+Boer+NK"%5BAuthor%5D), [Jarbandhan SV](http://www.ncbi.nlm.nih.gov/entrez/query.fcgi?db=pubmed&cmd=Search&itool=pubmed_Abstract&term="Jarbandhan+SV"%5BAuthor%5D), [de Graaf P](http://www.ncbi.nlm.nih.gov/entrez/query.fcgi?db=pubmed&cmd=Search&itool=pubmed_Abstract&term="de+Graaf+P"%5BAuthor%5D), [Mulder CJ](http://www.ncbi.nlm.nih.gov/entrez/query.fcgi?db=pubmed&cmd=Search&itool=pubmed_Abstract&term="Mulder+CJ"%5BAuthor%5D), [van Elburg RM](http://www.ncbi.nlm.nih.gov/entrez/query.fcgi?db=pubmed&cmd=Search&itool=pubmed_Abstract&term="van+Elburg+RM"%5BAuthor%5D), [van Bodegraven AA](http://www.ncbi.nlm.nih.gov/entrez/query.fcgi?db=pubmed&cmd=Search&itool=pubmed_Abstract&term="van+Bodegraven+AA"%5BAuthor%5D). Azathioprine use during pregnancy: unexpected intrauterine exposure to metabolites.Am J Gastroenterol. 2006 Jun;101(6):1390-2. [↑](#endnote-ref-2)
